# Bilateral Phrenic Nerve Palsy Associated With Neuralgic Amyotrophy

**DOI:** 10.7759/cureus.58069

**Published:** 2024-04-11

**Authors:** Wataru Shiraishi, Yuichi Murata, Yukiko Inamori, Ayano Matsuyoshi, Yusuke Nakazawa

**Affiliations:** 1 Neurology, Kokura Memorial Hospital, Kitakyushu, JPN; 2 Internal Medicine, Shiraishi Internal Medicine Clinic, Nogata, JPN; 3 Respiratory Medicine, University of Occupational and Environmental Health, Kitakyushu, JPN

**Keywords:** supine position, slurp, orthopnea, neuralgic amyotrophy, bilateral phrenic nerve palsy

## Abstract

Neuralgic amyotrophy (NA) is a multifocal inflammatory neuropathy accompanied by acute pain and muscle atrophy. NA commonly affects the upper extremities, but rarely affects the phrenic nerve. Here, we report a male with neck pain, orthopnea, difficulty sleeping in the supine position, and inability to slurp. His saturated oxygen level decreased from 97% to 86% in the supine position. His right shoulder showed muscle atrophy. Chest X-ray examination in the supine position and a nerve conduction study showed phrenic palsy. We diagnosed it as bilateral phrenic nerve palsy associated with NA. NA sometimes causes phrenic nerve palsy and may cause slurping difficulty.

## Introduction

Orthopnea is commonly seen in patients with heart failure or bronchial asthma. Heart failure-induced orthopnea results from fluid decomposition in the central circulation, causing pulmonary capillary pressure to increase and difficulty in breathing [1]. However, bilateral phrenic nerve palsy (BPP) can also trigger orthopnea. Causes of BPP include neurological diseases such as multiple sclerosis and amyotrophic lateral sclerosis, as well as complications of trauma and surgery, shrinking lung disease due to systemic lupus erythematosus, or idiopathic etiologies [1]. BPP is not a common condition, and a detailed investigation is needed to identify the cause [2]. The diagnosis of BPP is sometimes difficult to make, although a history of respiratory distress and chest X-rays in the upright and supine positions have been reported to be important in diagnosing BPP [3]. One Japanese report has described the chief complaint of inability to "slurp" as a characteristic finding of BPP [4], but this has not been recognized internationally. Here, we report a case of BPP who presented with neck and right upper extremity pain, followed by orthopnea. A detailed examination of the patient revealed atrophy of the right periscapular muscle, which led to a diagnosis of neuralgic amyotrophy (NA). NA is a rare disease of unknown cause that manifests with severe pain, followed by muscle atrophy and paresis. NA can sometimes damage the phrenic nerve, which can cause BPP [5,6], but is not well known and may be dismissed by clinicians. Our case was also accompanied by the chief complaint of an inability to slurp. Since slurping noodles or tea is common in Japanese culture, this may be a characteristic feature of BPP in Japan [4].

## Case presentation

A Japanese male aged in his 60s presented to our hospital with orthopnea, i.e., difficulty breathing in the supine position. He had a history of myocardial infarction one year earlier. His weight was initially 100 kg, which he had lost by dieting after the myocardial infarction. He did not experience any respiratory distress during sleep, even when he weighed 100 kg. His life history included occasional alcohol intake. He had smoked 20 cigarettes/day for 40 years, but had quit smoking one year earlier. He had no family members with similar symptoms. One month earlier, he developed a pain from the midline of the neck to the right shoulder (numeric rating scale: 9/10) without cause. At the same time, he began to have difficulty breathing when in a lying position. From the same period, it became difficult for him to slurp noodles or tea. He was admitted to another hospital with suspected myocardial infarction or heart failure, but his heart function was normal, so he was referred to our hospital one month after the onset of symptoms.

On physical examination, respiratory disturbance (oxygen saturation (SpO2) <90%) was noted only when the patient was in the supine position. The patient was 178 cm tall and weighed 90 kg. Vital signs included a blood pressure of 136/72 mmHg, pulse of 78 beats/min with a sinus rhythm, and body temperature of 36.5°C. The respiration rate was 20 breaths/minute. There was no skin rash or edema. The right periscapular muscle was atrophic. Upper arm circumference was right, 29 cm, and left, 29 cm, and forearm circumference was right, 27 cm, and left, 26.5 cm, showing no difference between the right and left arms. SpO2 was 97% in the standing and sitting positions, but decreased to 86% in the supine position.

Neurological examinations were as follows: intact cranial nerve, minimal muscle weakness in the proximal muscle of the right upper extremity, and a difference in manual muscle test of the deltoid and latissimus dorsi muscles between the right and left, with minimal weakness on the right, which was compatible for NA [5]. There was loss of tendon reflexes in the right upper extremity. There were no sensory deficits. Standing and walking were not affected, and the only limitation in the activities of daily living was dyspnea during sleep.

**Figure 1:**
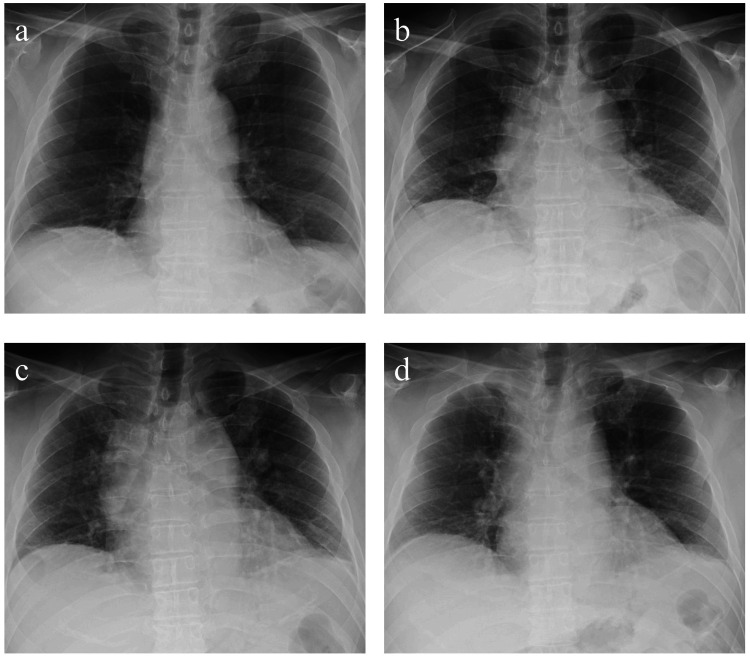
Chest X-ray of the patient on inspiration and expiration. In the upright position, diaphragmatic movements were seen on inspiration (a) and expiration (b), but in the supine position, these movements were absent (c: inspiration; d: expiration).

Blood tests revealed unremarkable electrolytes, thyroid function, liver enzymes, and renal function. Anti-nuclear antibody, anti-nuclear cytoplasmic antibody, angiotensin-converting enzyme level, anti-SS-A antibody, soluble interleukin-2 receptor, carcinoembryonic antigen, carbohydrate antigen 19-9, and rheumatoid factor were not elevated. The serum anti-aquaporin-4 antibody was negative. Blood gas analysis was performed in the supine position in room air, and pCO2 was 43.6 mmHg, but pO2 was 61 mmHg, showing hypoxemia. Spinal fluid was not examined because the patient could not lie still. Chest X-ray revealed a diaphragmatic movement in the upright position, but this movement was absent in the supine position (Figure 1).

**Figure 2:**
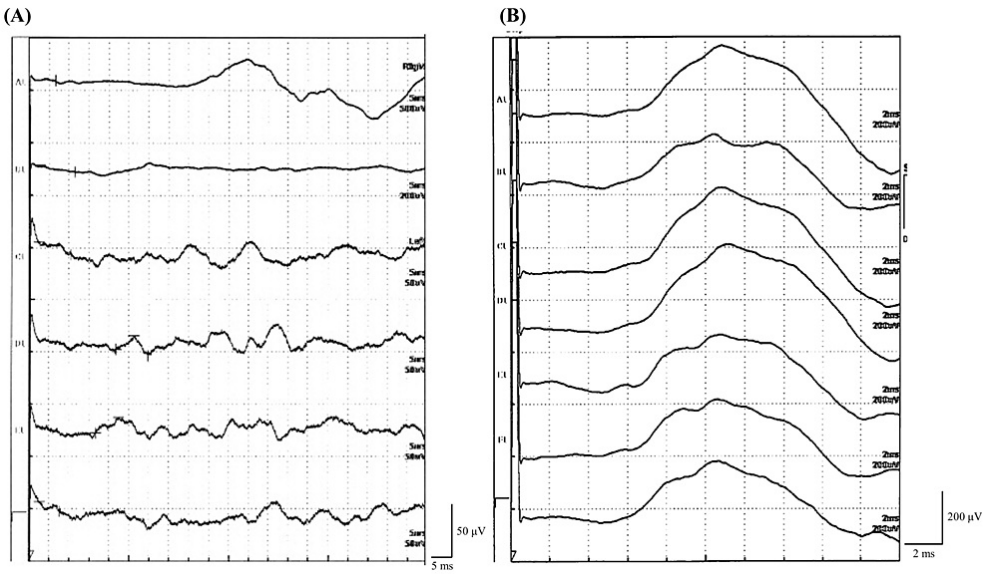
The patient's phrenic nerve conduction study findings. The patient's right phrenic nerve (A) showed a loss of amplitude as compared to the normal control (B). Note that the scale is magnified (A).

Nerve conduction studies of the right upper extremity were normal, and there was no demyelination or axonal involvement. However, the phrenic nerve showed a bilateral loss of response (Figure 2).

**Figure 3:**
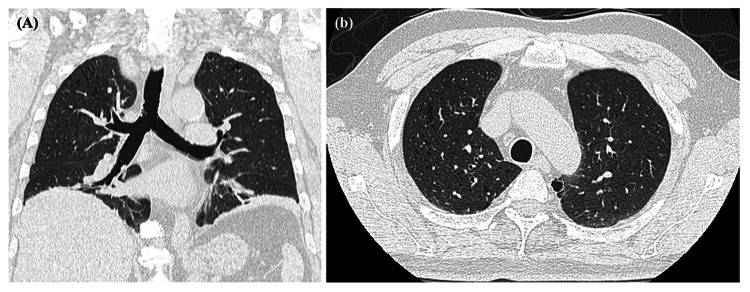
Chest CT of the patient. The chest CT showed no abnormalities in the pleural field. The CT did not include the periscapular muscles within the scanning window.

Needle electromyography was not performed because the patient refused. A respiratory function test showed a forced vital capacity (FVC) of 2.75 L in the sitting position, but only 0.53 L in the supine position. FVC fluctuation between sitting and supine positions in normal subjects is reported to be within 10% [7], but our patient's FVC was markedly decreased. The reason why his FVC in the sitting position was preserved is because the patient breathed through the abdominal muscles in the sitting position, as described in the "Discussion" section. Chest CT showed no vascular shunt, pulmonary congestion, or abnormalities in the pleural field (Figure 3).

We attempted an MRI of the head and spine, but could not perform it because the patient was unable to maintain a supine position.

Based on a medical history that started with severe pain and atrophy of the right periscapular muscle and according to the chest X-ray and electrophysiological examinations, a diagnosis of BPP associated with NA was made. In addition to introducing non-invasive positive pressure ventilation for respiratory failure, steroid pulse therapy (methylprednisolone 1,000 mg/day) was administered, followed by oral prednisolone (10 mg/day). His symptoms have shown gradual improvement; at approximately six months after onset, the patient still had dyspnea in the supine position and required non-invasive positive pressure ventilation for sleep. After nine months after onset, the patient was able to sleep without positive pressure ventilation. However, when sleeping without a respirator, the patient experienced a headache on awakening, suggesting residual respiratory failure.

## Discussion

NA was first described by Parsonage and Turner in 1948 [8] and was previously considered the same as brachial plexitis [9]. Recently, imaging and electrophysiological abnormalities have been identified in peripheral nerve trunks beyond the brachial plexus, so NA is now considered a multiple mononeuropathy rather than a brachial plexus neuropathy [10]. Therefore, due to NA, diaphragmatic nerve damage may also appear as a neuropathy other than the brachial plexus [5]. An interaction between mechanical compression and immunological mechanisms has been postulated as a cause of NA, but its precise etiology is unknown [11], and diagnostic criteria have not been established. Steroid administration and immunoglobulin in the early period of NA onset are reported to be effective [12,13]; however, in our patient, immunological treatment resulted in a slight improvement because it was started more than one month after symptom onset. Combined steroid pulse therapy and gammaglobulin therapy have been reported to be effective, but both were effective only in cases with early presentation [13,14]. In our case, gammaglobulin therapy was not administered because the patient was admitted long after a period from the symptom onset, the patient's job made long-term hospitalization difficult, and at that period, gammaglobulin was unavailable in Japan. Based on the typical clinical course and exclusion of other diseases, we diagnosed this case as NA, but an MRI could not be performed; thus, cervical myelopathy or radiculopathy was not sufficiently differentiated.

Concerning the relationship between NA and phrenic nerve paralysis, 7.6% of NA cases are associated with phrenic nerve palsy, of which 28% of cases (2.1% of all NA) are bilateral [5]. BPP often results in respiratory symptoms, and the critical diagnostic finding for phrenic nerve palsy is restricted diaphragmatic movement on inhalation and expiration on chest X-ray [6]; however, occasionally, as in our case, diaphragmatic nerve palsy is not apparent in the upright position, but is positive only in the supine position [15]. In patients with BPP on lying supine, it is reported that there is extrinsic compression of the thoracic cavity by the abdominal organs due to loss of caudal gravitational pull [16]. In our patient, this phenomenon was also indicated by a decrease in SpO2 from 97% in the sitting position to 86% in the supine position.

The mechanism of orthopnea resulting from BPP was as follows. In the standing or sitting position, in the exhalation phase, the diaphragm is elevated by contraction of the abdominal muscles, even though diaphragmatic contraction is lost. During the inhalation phase, the diaphragm can be lowered by gravity, and the lungs are ventilated by relaxation of the abdominal muscles. However, in the supine position, the patient cannot breathe using the abdominal muscles because of the loss of gravity assistance, leading to respiratory failure [3].

Previous cases of NA resulting in BPP and orthopnea are shown in Table 1.

Patients were mostly males in their 40s to 60s, and NA symptoms were rather right-sided. Most symptoms were dyspnea. Two cases were treated with immunotherapy, and none were treated with gammaglobulin. Some patients recovered within a few months, while others remained symptomatic for more than a year [6,17-20].

**Table 1:** Previous case reports of AN resulted in BPP and orthopnea. AN: amyotrophic neuralgia; BPP: bilateral phrenic palsy

Authors	Age	Sex	AN side	Symptoms	Treatment	Outcome
Holtbernd et al. [17]	55	Male	Both	Dyspnea, aphonia, impossible to cough	500 mg methylprednisolone for five days	After two months, phonation had markedly improved, but hoarseness was still present
Ikegami et al. [6]	67	Male	Both	Breathing difficulties at night	No particular treatment	Pulmonary function recovered to normal after seven years
Lahrmann et al. [18]	59	Male	Both	Severe dyspnea	No particular treatment	Relief of dyspnea after 18 months
	52	Female	Both	Severe nocturnal oxygen desaturations (<70%)	No particular treatment	Shortness of breath remained after 21 months
	52	Male	Right	Dyspnea that increased on effort	No particular treatment	Dyspneic on moderate effort after one year
Herbert et al. [19]	45	Male	Left	Shortness of breath worse on lying flat	Non-invasive ventilation	Symptom relief
Shinder et al. [20]	61	Female	Right	Severe dyspnea	Bilevel positive airway pressure	15 months, diaphragm function remained impaired
Our case	60s	Male	Right	Orthopnea	Steroid therapy	Gradual improvement

In clinical findings, our case presented with a characteristic medical history of a "slurping disturbance." The presence of being "unable to slurp noodles, water, or tea" has been reported as a characteristic medical history of BPP in Japan [4] because the habit of slurping food or drink is common in Japan [21]. The reason why BPP causes slurping difficulty is that the diaphragm is the most important respiratory muscle and is reported to be responsible for 2/3-3/4 of the change in tidal volume [22]. When it comes to rapid inhalation, hiccups can be a good example. Hiccups are spasms of the diaphragm caused by phrenic nerve excitation, and phrenic nerve blockade has been reported to halt intractable hiccups [23]. By a similar mechanism, we thought that BPP might prevent early inhalation with the diaphragm, leading to an inability to slurp. As such, an inability to slurp may be important for detecting BPP in Japan. This is the first English case report regarding BPP and slurping difficulty.

## Conclusions

We report a case of BPP caused by NA. Our patient had minimal weakness in the upper arms, so his medical history and chest radiographs taken in the supine position were helpful for diagnosis. In the supine position, respiratory support by the abdominal muscles was insufficient, and respiratory impairment due to BPP was exacerbated. In addition to a nerve conduction study of the phrenic nerve, chest X-ray examination in the supine position and respiratory function tests, as well as a history of being "unable to slurp," may be helpful in the diagnosis of BPP.
